# On the Modus Operandi of Quinine in Intermittent and Remittent Fevers

**Published:** 1849-09

**Authors:** D. Warren Brickell

**Affiliations:** New Orleans


### *On the Modus operandi of Quinine in Intermittent and Remittent Fevers.* By D. Warren Brickell, M. D., of New Orleans.

*Messrs Editors,*—In the July number of the "American Journal of the Medical Sciences," we find some very interesting "Notes of Hospital Cases," from the pen of Dr. H. Hartshorne of Philadelphia. Amongst others, he mentions some cases of intermittent and remittent fevers, treated by Dr. Gerhard, and volunteers the following deduction.

"The impression was formed, that of the two elements of fall fever—the febrile condition and the *miasm-cause*—it is the latter only that is addressed by the remedy in question. It arrests fever by acting as an antidote to the poison which produces it.

"Can such facts, then, point to its use with a similar intention in the exacerbations of other fevers, entirely different in cause, as the typhus and typhoid ? Certainly not."

Now, may we be allowed to ask of Dr. H. what this "*miasm-cause*" is ? What is its form, consistency, chemical composition ? Is it either visible or tangible ? Indeed, we should be most happy to have anything connected with its history, anything which is beyond the pale of disputation, anything veritably established. Moreover, such information being furnished us, we would be pleased to know the action of quinine on this "*miasm-cause*" out of the human body, either chemically or otherwise.

In the mean time, however, we will take it for granted that

*nothing* is as yet known of the nature of this supposed element, and venture a few humble animadversions on the deductions of the writer.

If, as we assert, we are all in total ignorance of the nature of the element, what right has the writer to imagine its presence in the system during an attack of intermittent or remittent fever? How very difficult is it frequently to detect the presence of, or to remove foreign bodies, *causes* of disease, even where they possess all those physical characteristics which tend to render them evident in every sense of the word! Again, remove the foreign body, a splinter, a nail, &c., or allow nature herself to perform the task; days elapse, during which time the patient appears to be doing well; suddenly, however, tetanus supervenes; the life of the patient is threatened, notwithstanding the removal of the original *cause.* Shall we still suppose the presence of this cause, and resort to the knife, its only antidote; or shall we address our remedies to the symptoms of the patient? Suppose a man is laboring under what is called "coup de soleil;" in this case the cause of the disease, although subtle, is more evident than the "*miasm-cause*;" but do we address our remedies to the sun? Have we any right to suppose the existence of the sun or its causative emissary in the body of the patient?

But let us approach the bed-side of a patient. We find him with hot and dry skin, accelerated pulse, indeed all the symptoms characterising that state ordinarily denominated *fever.* 'Tis a case of remittent fever, and we find him in the midst of an exacerbation. He tells us he is six days from Wilmington, N. C.; that he was was taken sick the day after leaving said port; has had an exacerbation of fever every day during five days. We administer 15 to 30 grains of quinine; in three hours his pulse is very much reduced; his skin is bathed in a free perspiration; no pain; sleeps quietly; we all agree that this change of condition is attributed to the quinine.

Now the question arises, whether the remedy acted on the *cause* of the disease, (of which we are most profoundly ignorant,) or on the man himself. If on the *cause*, we should be highly gratified to be furnished with the data for such a conclusion, together with something explanatory of the change produced in the condition of the man—as the reduction of the pulse, free perspiration, &c. Surely the writer will not contend that the antidotal effect of the quinine on the "*miasm-cause*" within the man, has any agency in effecting this happy change. For our own part, the phenomena consequent on the exhibition of quinine, have always impressed us with the belief that the agent acts on the man himself, and it must be something more powerful than a mere supposition, a theory, that can convince us to the contrary. When we apply sinapisms to the cold extremities of a patient laboring under congestive fever, (a so-called *miasmatic* fever,) and a rubefacient effect is produced, thereby in a great degree improving the condition of the man, we can readily conceive of the action of the remedy on the part to which it has been applied, but it must be a great stretch of the imagination to think for a moment that the *cause* of the disease has been acted on by the mustard.

But quinine sometimes *fails* to cure fever, or, in Dr. H.'s view of the question, to *antidote* the *cause*. How does the writer account for this? Such cases, too, will often yield very readily to arsenic; sometimes to opium; sometimes to the purgative plan of treatment. We have seen cases of mild remittent fever cured by the application of the cold *douche*. Are all these remedies antidotes to the "*miasm-cause*"? &c.

However, we think the Dr. abandons his own position even before the sound of the enemy's trumpet reaches his ear. In one sentence he says, "It (quinine) arrests fever by acting as an antidote to the poison which produces it." And the second sentence after this, he says, "The writer saw no facts, in any case of its administration, to justify any other appellation for its effects than those of tonic and nervous stimulant."

"Tonic and nervous stimulant" of what? Not of the "*miasm-cause*;" but of the patient to whom the remedy was administered. He acknowledges this "tonic and nervous stimulant" effect; and yet asserts that it is the "*miasm-cause*," only, which is addressed by the remedy. To us all this seems "confusion worse confounded." Despite the careful "operation of displacement" to which the author has subjected the "*miasm-cause*," this imaginary something—or rather, nothing—is found to be (in the ordinary phraseology of the day,) about "as clear as mud."

When the writer has personally tested the efficacy and modus operandi of the "large quantities" of quinine administered by

"some southern practitioners," the latter will more willingly listen to his speculations concerning the probable causes of the sedative action on the pulse, whether it is "cerebral oppression, analogous to narcotism, or the *antidotal* influence above spoken of." He acknowledges that he has never administered the medicine in these "large quantities," and whilst we would most earnestly recommend the practice, we must contend that his present deductions are, to say the least of them, premature.

Whether quinine should be administered in typhus and typhoid fevers "with a similar intention,"—its antidotal influence over the "*miasm-cause*,"—we will not pretend to argue; perhaps the causes of these fevers—typhus and remittent—are totally different. One thing, however, we will venture to say; if the writer will administer 30 or 40 grains of quinine, combined with a grain or two of opium, to cases of typhus and typhoid fevers in the *commencement* of the attack, we think he will find the agent quite as efficient in checking these fevers as it is in checking the so-called *miasmatic* fevers. These results we have repeatedly witnessed, but we have never yet concluded that quinine is an antidote to the *cause* of typhus fever.

